# VS-4718 enhances apoptosis induced by low-dose carfilzomib and overcomes carfilzomib resistance in *PSMB5*-mutated proteasome inhibitor resistant multiple myeloma

**DOI:** 10.1038/s41598-026-43205-4

**Published:** 2026-03-16

**Authors:** Ellen Leich-Zbat, Sofia Catalina Heredia-Guerrero, Marietheres Evers, Thorsten Stühmer, Tina Grieb, Hilka Rauert-Wunderlich, Ralf C. Bargou, Andreas Rosenwald, Manik Chatterjee, Daniela Brünnert

**Affiliations:** 1 Institute of Pathology, University of Würzburg, 97080 Würzburg, Germany; 2 Comprehensive Cancer Center Mainfranken, University Hospital of Würzburg, Würzburg, Germany; 3 Experimental Tumor Immunology, Department of Obstetrics and Gynecology, University Hospital of Würzburg, Würzburg, Germany

**Keywords:** Multiple myeloma, Proteasome inhibitors, PYK2, VS-4718, Carfilzomib, Proteasome inhibitor resistance, Cancer, Drug discovery, Oncology

## Abstract

The introduction of proteasome inhibitors (PIs) into multiple myeloma (MM) treatment has substantially improved therapeutic options and survival rates. However, the development of resistance to PIs as well as serious side effects of PI treatment continue to justify the search for effective, less burdensome combination therapies. Thus, we investigated the therapeutic potential of VS-4718 (dual pPYK2- and pFAK-inhibitor) alone and in combination with the PI carfilzomib (carf) in parental human MM cell lines (pHMCLs) and PI triple-resistant HMCLs (rHMCLs). VS-4718 reduced the viability in a concentration dependent manner in all pHMCLs and the response was independent of PYK2 expression or activation. A more than additive impact of the combination therapy on survival, measured by annexin V-FITC/PI staining, was observed in 5/7 pHMCLs. Titration experiments showed that VS-4718 in combination with low and sublethal doses of carf had a specific anti-tumor effect on pHMCLs but hardly affected peripheral blood mononuclear cells. In rHMCLs, addition of VS-4718 to carf re-sensitized cells to carf and revealed a more than additive reduction in cell survival. These findings suggest that VS-4718 together with low doses of carf could be an effective and low-toxic combination in MM, including PI-resistant, relapsed/refractory MM.

**Supplementary Information:**

The online version contains supplementary material available at 10.1038/s41598-026-43205-4.

## Introduction

Multiple myeloma (MM) is characterized by multifocal clonal expansion of terminally differentiated plasma cells in the bone marrow. Due to the introduction of the first- and second-generation proteasome inhibitors (PIs) bortezomib (btz), carfilzomib (carf) and ixazomib (ixa), immunomodulatory drugs (IMiDs), immunotherapies (monoclonal antibodies, bispecific antibodies and CAR-T cells) into myeloma therapy, the median survival time of MM patients has increased to over 10 years^1,2^. Despite this success, however, most patients develop resistance to PIs, and some suffer from severe side effects of PI treatment^3,4^.

One of the most important questions in myeloma therapy is how this resistance can be overcome and how severe side effects induced by drugs such as carf could be reduced, particularly in fragile patients with relapsed/refractory MM.

Several PI resistance mechanisms have been identified^3,5^. Some include generalized adaptations to the stresses of cancer growth and treatment, such as hypoxia-induced metabolic adjustments and expression changes of transmembrane carriers^6^. Mechanisms that are more specifically linked to PI resistance include the aberrant expression of ubiquitine-proteasome pathway components and the occurrence of mutations in the proteasome 20 S subunit beta 5 (*PSMB5)*^7–9^. These mutations specifically inhibit the binding of PIs to the proteasome through steric changes at the PI binding site^5,7^.

Due to its high genetic heterogeneity and the scarcity of targetable driver events, few individualized treatment concepts are currently available for MM. Nevertheless, several oncogenic drivers have been identified whose potential as therapeutic targets requires further investigation.

One of these is the focal adhesion kinase protein tyrosine kinase 2 beta PYK2 (also known as PTK2B), which plays a role in many human cancers^10^. It is frequently overexpressed in MM cells compared to normal plasma cells^11^ and it is associated with survival signaling and tumor growth in MM in vitro and in vivo^11–13^. Dual FAK/PYK2 inhibitors are currently being tested in clinical trials for various types of cancer^14^, including an ongoing clinical trial with NF2 mutated refractory cancers, involving solid cancers, lymphoma and multiple myeloma (NCT04439331, currently *n* = 30). The dual pFAK and pPYK2 inhibitor VS-4718 was tested in the Pediatric Preclinical Testing Program (PPTP) where it was well tolerated^14^ and a phase I clinical trial of VS-4718 in combination with cytostatic agents (paclitaxel and gemcitabine) for advanced prostate cancer revealed good anti-tumor effects^14^.

The inhibition of pPYK2 with either VS-4718 or VS-6063 and 5 nM btz overcame hypoxia-induced btz resistance in two HMCLs (MM.1 S, H-929) and in a subcutaneous mouse-model with H-292 cells^13^. Likewise, a combination of either VS-4718 or VS-6063 with 5nM carf reduced the metabolism/viability in these two HMCLs more effectively than carf alone^13^. These are promising findings which warrant further investigation of the therapeutic potential of dual pPYK2 and pFAK inhibitors for cell death induction in MM in general as well as in PI-resistant MM.

Specifically, this study aimed to investigate if (1) VS-4718 has an impact on survival in a range of genetically different MM cell lines (2) it is possible to reduce carf to concentrations with very little toxicity without losing the additional killing effect in combination with VS-4718 (3) the addition of VS-4718 to carf allows to overcome PI-specific resistance in PI-triple-resistant *PSMB5* mutated cells and (4) the response to VS-4718 is dependent on the levels of activated PYK2 and/or FAK.

## Materials and methods

### Cell lines and culturing conditions

The parental human multiple myeloma cells (pHMCLs) (L-363, AMO-1, U-266, KMS-12-BM, JJN.3 (DSMZ, Braunschweig, Germany) KMS-11 (Japanese Collection of Research Bioresources (JCRB)), MM1.S (LGC Biolabs, Wesel, Germany)) which were derived from MM patients at different clinical stages and represent different molecular profiles^15,16^ were cultured in RPMI-1640 medium supplemented with 10% FBS, 2 mM L-glutamine and 1 mM sodium pyruvate^8^. The resistant HMCLs (rHMCLs) L-363R350Ixa and MM.1SR180Ixa, which also showed a cross-resistance to the proteasome inhibitors bortezomib and carfilzomib, were previously generated by us and were cultured accordingly^8^. However, to keep the cells resistant, 1–2 days after thawing, 200 and 75 ng/ml ixa were added to the L-363R350Ixa and MM.1SR180Ixa cells, respectively, until two weeks before the experiments started.

Peripheral blood mononuclear cells (PBMCs) were obtained from buffy coats of healthy donors (Klinische Transfusionsmedizin und Hämotherapie, Universitätsklinikum Würzburg) and cultured as previously described^17^. The study was conducted in accordance with the Declaration of Helsinki. Institutional permission and oversight of human sample handling was reviewed by the Ethics Committee of the University of Würzburg (76/13, 146/23_am).

### Inhibitors

All inhibitors were dissolved in H_2_O-free DMSO and stored at the indicated stock concentrations and temperature. Carf, ixa and VS-4718 were purchased from Selleck Chemicals (Houston, Texas, USA). Working solutions were always prepared freshly by further dilution of the stock solutions in cell culture medium. Suitable concentrations of carf were determined individually for each MM cell line.

### Metabolism/viability and survival analysis

#### MTT assay

Following incubation with 0.1–10 µM VS-4718 for 96 h, metabolism/viability was measured according to the protocol described in^18^.

#### Annexin V-647/propidiumiodide staining and FACS analysis

For FACS analysis 5 × 10^5^ cells were seeded in a 6-well plate and incubated with cell culture medium and different concentrations of VS-4718 and carf alone and in combination for 72 h. Cells were then stained with annexin V-647)/propidiumiodide (annexin V/PI) and measured by FACS (BD FACS Canto II, BD Biosciences) as previously described^8^.

For the graphical representation, the annexin V/PI negative fraction was calculated relative to that in the DMSO-treated control sample (% annexin V/PI neg. cells rel. ctrl.).

### Western blot analysis

Western blots were accomplished as previously described^19^ using antibodies from Cell Signaling Technologies (Danvers, MA, USA) against the following targets: PYK2 ((H364), #3090, 1:1000), pPYK2 ((Y402) #3291, 1:2000), FAK ((D2R2E) #13009, 1:1000), pFAK ((Y397) #3283, 1:1000), CASP9 (#9508, 1:1000 and PARP-1 ((46D11), #9532,1:1000). To detect pFAK the high sensitivity enhanced luminol-based chemiluminescent substrate solution WESTAR SUPERNOVA XLS3,0100 from Cyanagen was used.

To compare the expression and activation of proteins before and after treatment with VS-4718, carf and VS-4718 + carf, cells were transiently cultured in a 6-well format and treated with DMSO or the specified drugs for 24 h.

Image J analysis was performed to analyze the GAPDH-normalized expression and activation levels of the different protein markers compared to the DMSO control.

### Statistics

To evaluate the differences in expression/activation and the differences in survival one-way and two-way ANOVA (with Geisser-Greenhouse correction) were performed using GraphPad-Prism 10.4.2. Multiple comparisons were accomplished using Tukey’s multiple comparison tests.

## Results

### VS-4718 effectively reduced metabolism/viability and induced PARP-1 cleavage in various MM cell lines with different levels of PYK2 and FAK expression/activation

**Fig. 1:**
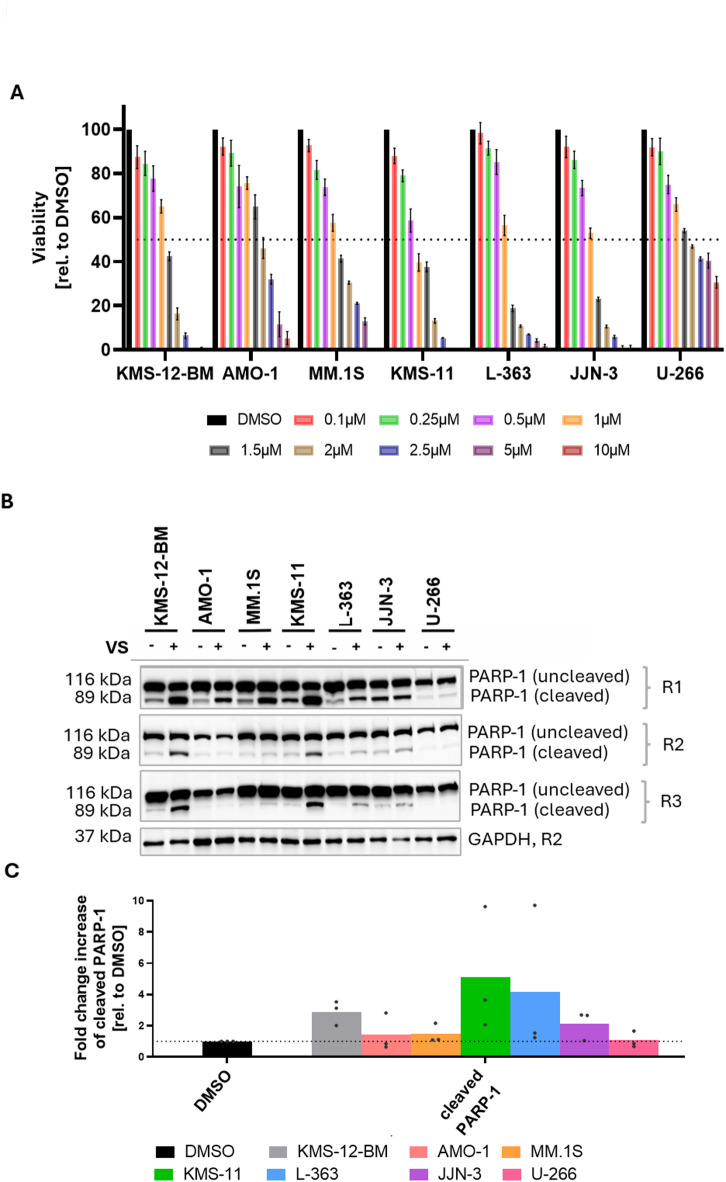
Impact of VS-4718 on viability and PARP-1 cleavage in seven MM cell lines. (**A**) MM cells were treated with different concentrations of VS-4718 (0.1–10 µM) for 96 h and viability subsequently measured by MTT assay in three independent experiments. (**B**, **C**) MM cells were treated with 2 µM VS-4718 and PARP-1 cleavage determined by Western blot analysis in three independent experiments (R1, R2, R3). (**B**) Only one representative GAPDH (R2) was shown in this Western blot picture. (**C**) Image J analysis was performed to analyze the GAPDH-normalized expression levels of cleaved PARP-1 compared to the DMSO control. Bars represent the mean ± SEM of three independent experiments. Western blots were cropped. For uncropped Western blots see supplementary information.

The titration of VS-4718 (0.1–10 µM) in seven MM cell lines (L-363, AMO-1, U-266, KMS-12-BM, JJN.3, KMS-11, MM.1 S) with various genetic backgrounds^15,20^ and different levels of PYK2 and FAK expression/activation (**Figure ****S1**) showed that a concentration of 1 µM VS-4718 was sufficient to distinctively reduce the metabolism/viability in all MM cell lines tested (Fig. 1A). At 2 µM, viability was reduced by more than 50% in all MM cell lines (Fig. 1A). KMS-11, L-363 and JJN-3 cells showed the best responses in this assay at concentrations of 1–2 µM (Fig. 1A).

Moreover, PARP-1 cleavage has been observed repeatedly (in three independent rounds) after 24 h incubation in 3/7 HMCLs using 2µM VS-4718 (Fig. 1B **+ C**). AMO-1 and JJN-3 showed PARP-1-cleavage in only one and two out of three independent rounds upon VS-4718 treatment, respectively (Fig. 1B **+ C**).

### The combination of VS and carf entailed a higher than additive reduction of survival in five out of seven MM cell lines tested

**Fig. 2:**
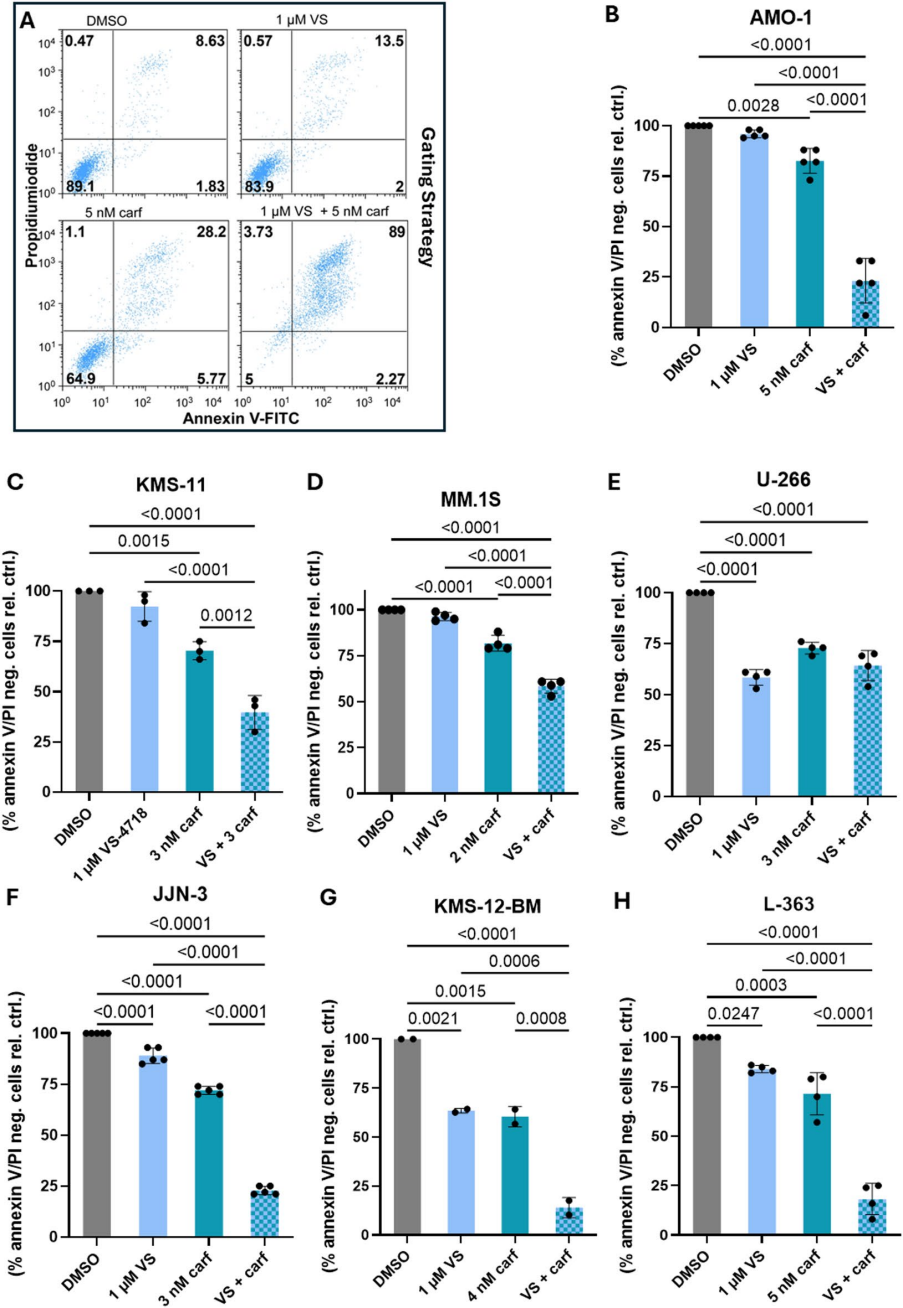
Impact of VS-4718 alone and in combination with carf on survival in seven MM cell lines. (**A**–**H**) MM cells were treated with 1 µM VS and moderate concentrations of carf alone and in combination. Suitable concentrations of carf were determined individually for each MM cell line. Survival was determined by Annexin V-647/PI staining and subsequent FACS analysis. (**A**) An experiment with the AMO-1 cell line was used to illustrate the gating strategy employed here and in further FACS experiments. (**B**–**H**) For the graphical representation, the annexin V/PI negative fraction was calculated relative to that in the DMSO-treated control sample (% annexin V/PI neg. cells rel. ctrl.). Bars represent mean ± SD of ≥ three independent experiments, except for KMS-12-BM (2 rounds). *P*-values were determined in an ordinary one-way ANOVA using Tukey’s multiple comparison test and depicted only for *p* < 0.05. Only statistical comparisons between DMSO control and treated cells as well as single drugs vs. drug combinations were depicted to focus on the relevant comparisons.

To investigate combination effects of VS-4718 and carf on cell death induction in the seven HMCL used herein, we treated the cells with 1 µM VS-4718 and “moderate” (i.e. within the EC_10_-EC_50_ range) doses of carf. The effect on survival as determined by annexin V/PI staining was at least additive (for 6/7 HMCLs tested) or even stronger (for 5/7 HMCLs tested, Fig. 2A-H).

### The combination of higher concentrations of VS-4718 with low concentrations of carf reduced survival of MM cells efficiently

**Fig. 3:**
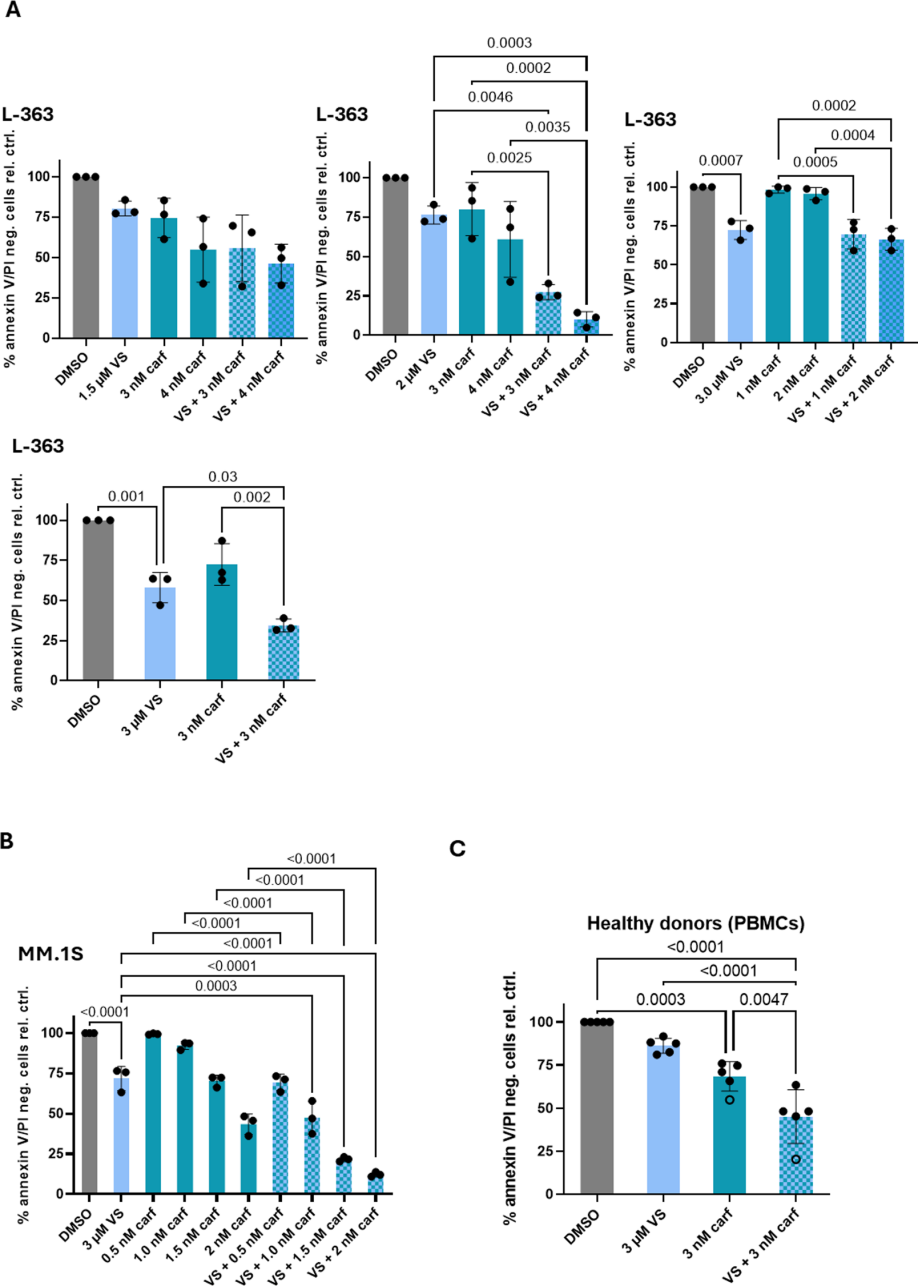
Titration of VS-4718 and carf in two MM cell lines. To find an effective and low toxic combination of carf and VS-4718, these drugs were titrated in (**A**) the cell line L-363 in different combinations of VS-4718 (1.5-3 µM) with carf (1–4 nM) starting with low doses of VS-4718 and high doses of carf, followed by high doses of VS-4718 with low doses of carf. 3 µM VS-4718 with 3 nM carf was determined as optimum in this test series. (**B**) 3 µM VS-4718 was then taken for the titration with different doses of carf (0.5-2 nM) in MM.1 S, taking previously reported carf concentrations as reference (Brünnert, Kraus et al. 2019). (**C**) 3 µM VS-4718 and 3 nM carf were tested in primary PBMCs. (**A**–**C**) Survival was determined by Annexin V-647/PI staining and subsequent measurements with a BD FACS Canto II from BD Biosciences. Bars represent the mean ± SD of three independent experiments. *P*-values were determined in an ordinary one-way ANOVA using Tukey’s multiple comparison test and only depicted for *p* < 0.05. The depiction of statistical comparisons was furthermore limited to DMSO vs. VS-4718 and single drugs (VS-4718 and carf) vs. drug combinations to focus on the most relevant results.

Because carfilzomib exhibits considerable toxicity at higher concentrations, we sought to reduce its dose as much as possible while preserving maximal effects on survival. To this end, we titrated both drugs individually (VS-4718: 1.5-3.0 µM, carf: 1–4 nM) and their combinations using L-363 cells (**Figure S2**, Fig. 3A**)**.

We started with the lowest doses of VS-4718 and the highest doses of carf, followed by a down-titration of carf and increased concentrations of VS-4718. To be able to compare the effect of VS-4718 on both cell lines used, the same VS-4718 concentration was used with MM.1 S cells. However, the appropriate concentration of carf needed to be downscaled for the more carf-sensitive cell line MM.1 S (Fig. 3B), taking previously published results as reference^8^.

Treatment with 3 µM VS-4718 entailed a notable increase of cell death (annexin V/PI positive cells) for both L-363 (~ 40%) and MM.1 S (~ 30%).

The addition of only 3 nM carf in L-363 cells and 1.5 nM carf in MM.1 S cells, which, by itself had only little impact on survival, effectively reduced cell survival by 70–80% in combination with VS-4718 (Fig. 3A, B).

In contrast to MM cells which were sensitive to 3 µM VS-4718 and highly sensitive to the combination of 3 µM VS-4718 and 3 µM carf, 3 µM VS-4718 alone had no significant impact on the survival of primary PBMCs and the combination with carf reduced cell survival by only 50% in healthy donors (Fig. 3C).

### VS-4718 overcomes resistance in PSMB5-mutated PI triple-resistant MM cells

Finally, we tested whether 3 µM VS-4718 alone or in combination with low and sublethal doses of carf would reduce survival and overcome the PI-resistance in ixa-resistant and *PSMB5*-mutated MM cell lines, which also have a proven resistance against btz and carf^8^. Here, we used the L363R350Ixa cell line harboring a heterozygous *PSMB5* mutation and MM1.SR180Ixa cells which are affected by a hemizygous *PSMB5* mutation, a deletion of the other allele, an upregulation of PSMB5 and β5-activation^8^.

**Fig. 4:**
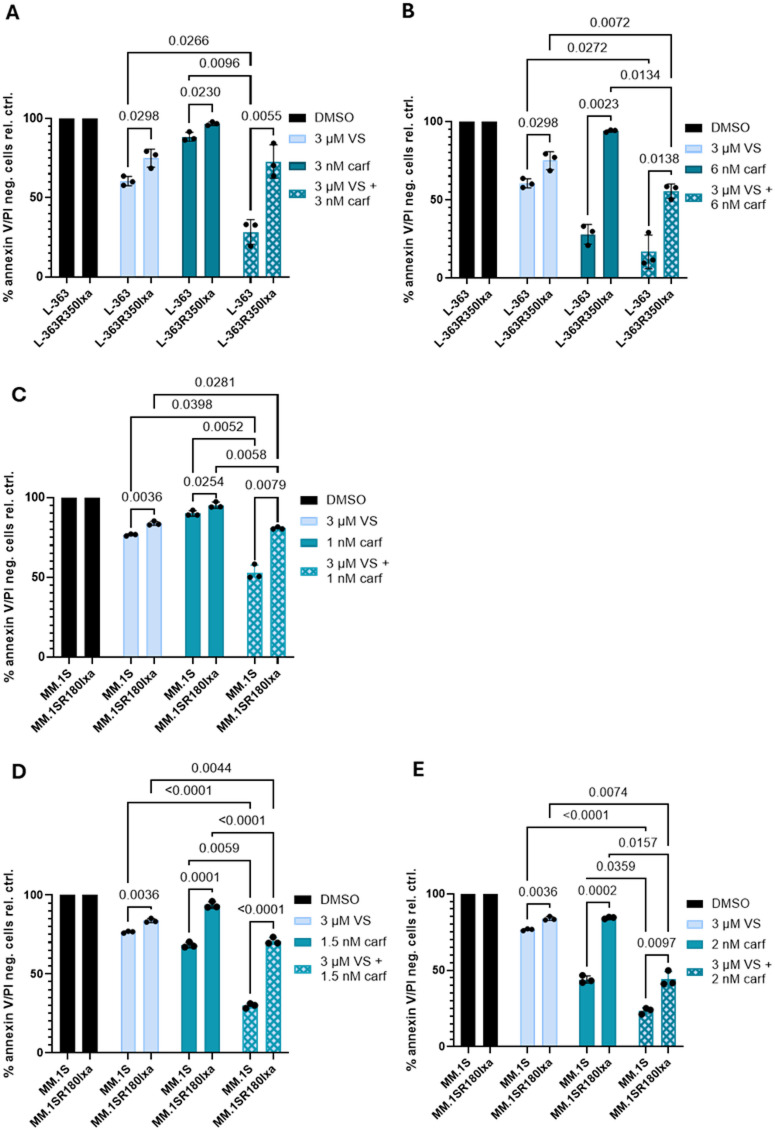
Potential of VS-4718 to enhance the effects of carf in parental and resistant HMCLs. (**A**) L-363 and L363R350Ixa cells were treated with 3 µM VS-4718 and 3 nM carf, or (**B**) 6 nM carf. (**C**) MM.1 S and MM1.SR180Ixa cells were treated with 3 µM VS-4718 and 1 nM carf, (**D**) 1.5 nM carf, and (**E**) 2 nM carf. Survival was determined by Annexin V-647/PI staining and subsequent measurements were performed with a BD FACS Canto II from BD Biosciences. Bars represent the mean ± SD of three independent experiments. *P*-values were determined in a two-way ANOVA using Geisser-Greenhouse correction and a Tukey’s multiple comparison test and only depicted for *p* < 0.05. To focus on the most relevant findings, the depiction of statistical comparisons was limited to comparisons of single drugs vs. drug combinations and parental vs. PI-resistant cell lines.

Titration of carf in pHMCL and rHMCL (3–12 nM in p/r L-363 and 0.5-3 nM in p/r MM.1 S) confirmed the previously published resistance of rHMCL to carf^8^, Fig. 4A-E, **Figure S3**). Treatment with 6 nM carf reduced the survival of L-363 cells by approximately 75%, but only by ~ 5% regarding L363R350Ixa cells (Fig. 4B, **Figure S3**). At 12 nM carf approximately 85% of L363R350Ixa cells were still alive (**Figure S3**). The resistance of MM1.SR180Ixa cells against carf, when compared to MM.1 S cells, was less pronounced, but still clearly visible (Fig. 4C-E), as previously reported^8^. In contrast to the situation described in a comparison of hypoxia-resistant and normoxia MM.1 S and H-929 cells^13^, the addition of VS-4718 had a slightly stronger effect in parental HMCLs compared to PI triple-resistant MM.1 S and L-363 cells (Fig. 4A-E).

Although concentrations of 6, 8 and 10 nM carf reduced survival of L-363R350Ixa cells by only ~ 5, ~10 and ~ 15% (**Figure S3**), the combination of VS-4718 and 6 nM carf led to ~ 45% cell death (Fig. 4B). 3 µM VS-4718 and 2 nM carf, when used alone, each led to cell death of about 18% of MM.1SR180Ixa cells (Fig. 4E). Conversely, the combination of 3 µM VS-4718 with 2 nM carf achieved a cell death rate of ~ 60%, which is ~ 25% more than simple additivity of the effects of the two compounds (Fig. 4E).

**Fig. 5:**
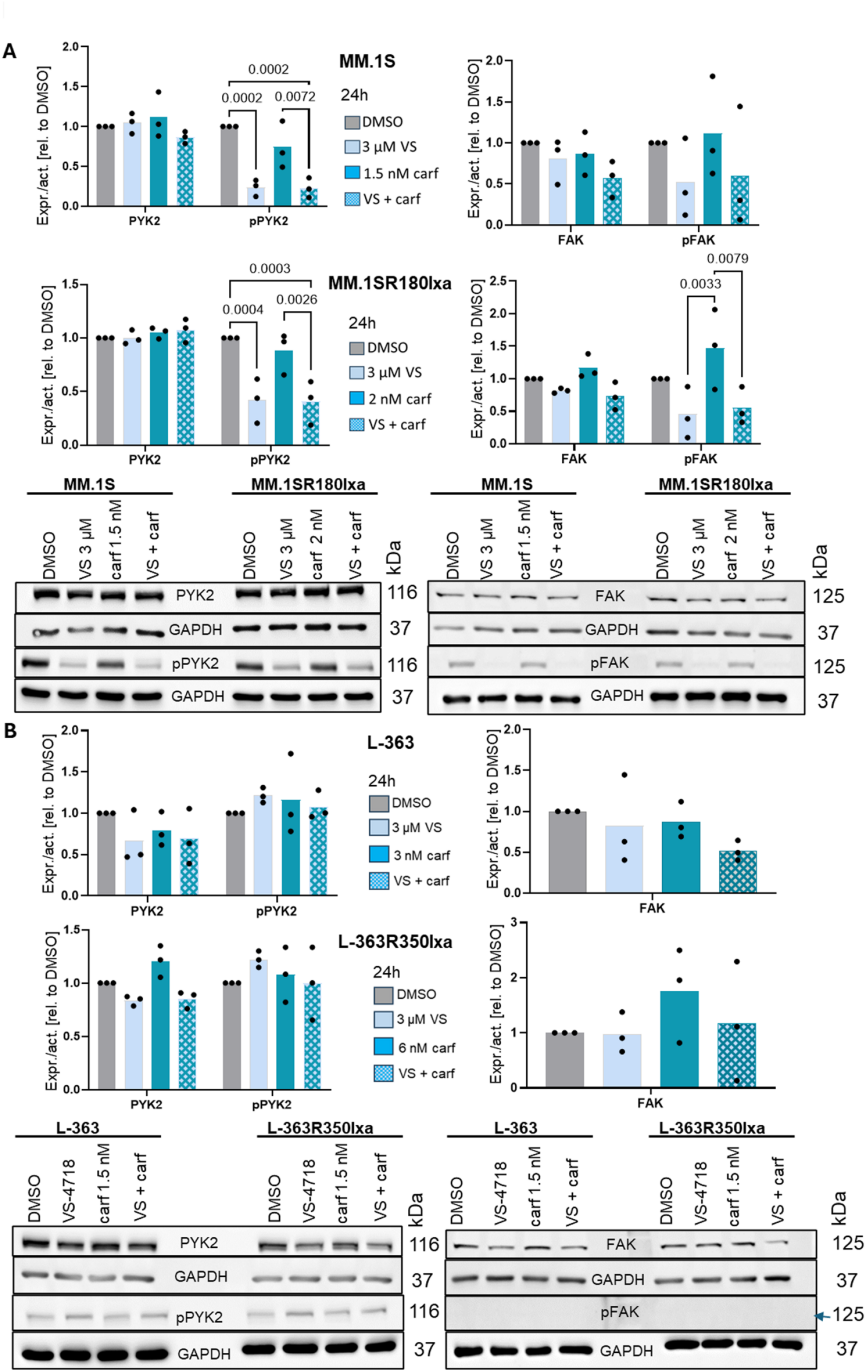
Western blot analysis to determine the PYK2 and FAK expression and activation status before and after inhibition with VS-4718 and carf alone and in combination. (**A**) MM.1 S cells treated with 3 µM VS-4718 and 1.5 nM carf alone and MM1.SR180Ixa cells with 3 µM VS-4718 and 2 nM carf for 24 h (**B**) L-363 cells were treated with 3 µM VS-4718 and 3 nM carf and L363R350Ixa cells with 3 µM VS-4718 and 6 nM carf for 24 h. (**A**,** B**) Image J analysis was performed to analyze the GAPDH-normalized expression and activation levels of (p)PYK2 and (p)FAK compared to the DMSO control. Bars represent the mean ± SD of three independent experiments. Western blots were cropped. For uncropped Western blots see supplementary information. *P*-values were calculated with a two-way ANOVA and an ordinary one-way ANOVA (for FAK in L-363 and L-363R350Ixa) using the Tukey’s multiple comparison test with multiple comparison test and only depicted for *p* < 0.05.

Retrospective analysis of Western blots of cells drug-treated for 24 h and previously used for signaling analysis (see Fig. 5) showed strong increase in PARP-1 cleavage for the VS+carf combination in L-363 and L-363R350Ixa cells (**Figure S4**), thus underpinning increased induction of apoptosis in the drug combination. For MM.1 S cells the effect in the combination barely exceeded that of VS treatment alone, but the timing for the harvest of these slower growing cells may have been less suitable.

In summary, carf concentrations that had only little cytotoxic effects in *PSMB5*-mutated and PI triple-resistant MM cells, became significantly more effective in combination with VS-4718.

### VS-4718 impacts viability and survival independent of PYK2 and FAK expression and activation levels

Since VS-4718 targets FAK and PYK2 by blocking their phosphorylation (pFAK and pPYK2)^14^, we also aimed to investigate the expression and activation of these (phospho-)proteins in the two pHMCL and their corresponding rHMCL before and after inhibition with VS-4718.

According to the observations made in previous experiments (Figs. 2, 3 and 4), we chose 3 µM VS-4718 and carf concentrations that only showed a little effect alone, but a strong effect in combination with VS-4718 in both parental and resistant HMCL (**Figure S3)**, pL-363: 3 nM carf, L-363R350Ixa: 6 nM carf, pMM.1 S: 1.5 nM carf, MM1.SR180Ixa: 2 nM carf).

In MM.1 S and the corresponding resistant cell line MM.1SR180Ixa, PYK2 and FAK activation was reduced by the addition of VS-4718 (Fig. 5A) and the expression levels of FAK and PYK2 remained unaffected in all cell lines (parental and resistant HMCL) (Fig. 5A **+ B**), as expected by the described mode of action for this drug^21^. In contrast, no reduction in the activation of PYK2 was observed in L-363 (Fig. 5B), although the effect on survival by adding 3 µM VS-4718 was clearly stronger in L-363 and L-363R350Ixa compared to MM.1 S and MM1.SR180Ixa (Fig. 4A **+ B**). Moreover, despite the use of a high-sensitivity ECL solution, pFAK was not detectable in L-363 and the corresponding resistant cell line L-363R350Ixa in this experiment (Fig. 5B).

The stronger effect of VS-4718 on survival in L-363 and L-363R350Ixa compared to MM.1 S and MM.1SR180Ixa is also reflected by an overall increased PARP-1 cleavage, which is especially prominent in combination with carf (**Figure S4**).

In summary, these observations and the observations made in seven HMCLs (**Figure ****S1**) suggest that the response to VS-4718 is not related to the level of PYK2 and/or FAK activation.

## Discussion

Proteasome inhibitors have clearly improved survival times for MM patients and are now an integral part of MM therapy. Carf, a second-generation PI, which has also demonstrated activity in btz-resistant MM, is often given to MM patients with relapsed refractory disease^5^. However, over time, MM patients inevitably develop resistance, and dose-limiting toxicities of PIs (e.g. carf-induced heart toxicities) somewhat curb their potential utility^3^. CAR T-Cell therapies also turned out to be very effective and seem to be another breakthrough in MM therapy. However, these therapies also have severe side effects (e.g. cytokine release syndrome and immune deficiency)^22^.

Therapies directed against specific targets, as are known from solid cancers, are rare in MM. The BRAF/MEK inhibitor combination therapy is an exception, as it can be applied in approximately 4% of patients with a BRAF V600E mutation^23–25^.

PYK2, a focal adhesion kinase, which can be activated by e.g. receptor tyrosine kinases was discovered to play an important oncogenic role in MM and applying dual pFAK and pPYK2 inhibitors to MM cells or a xenografted mouse model with injected MM.1 S cells revealed a notable effect on survival in vitro and in vivo^11–13^.

In the current study, a significant effect on the viability of MM cells was also observed in a large number of HMCLs (*n* = 7) with different mutation profiles^15,20^. It is noteworthy that the genes affected by these mutations also included potential PYK2 activators (e.g., ITGB, ITGA, EGFR) or PYK2 effectors (e.g., RAS, RAF, MEK, PI3K)^15^ and that the cell lines in the current study showed different expression and activation of FAK and PYK2.

Since neither the occurrence of mutations nor the degree of PYK2 or FAK activation appear to influence the tumor-inhibiting effect of VS-4718, this inhibitor could be a suitable drug for this highly heterogeneous entity.

Of note, while PYK2 expression is higher in primary MM cells compared to normal plasma cells, FAK expression is reduced in primary MM compared to the corresponding non-tumor cells and the activation of FAK was not even detectable by Western blotting in MM cell lines^11^. Given that oncogenes are supposed to be overexpressed, it might be more likely that PYK2 plays the central oncogenic role among the two focal adhesion kinases that are targeted by VS-4718 in MM. Nevertheless, we also included FAK in our investigations and used a high-sensitivity ECL solution which revealed slight but clear pFAK signals in at least 5/7 cell lines.

Another recent study used two cell line models and a subcutaneous mouse model with the MM cell line H-929 to demonstrate that the inhibition with dual pFAK and pPYK2 inhibitors in combination with btz and carf overcomes hypoxia-induced PI resistance^13^.

Since the inhibition of pFAK and pPYK2 with such inhibitors in combination with PIs seems to have great potential in MM therapy, we aimed to further explore a combination of VS-4718 with carf in the same seven HMCLs that were used for the viability assays. As already observed in the viability assay, all HMCLs showed a considerable reduction in survival upon the treatment with VS-4718 and a combination with moderate doses of carf revealed a higher than additive reduction in survival in all but the HMCL U-266, irrespective of their reported mutation status^15,20^, and irrespective of their PYK2 and FAK expression/activation status.

VS-4718, which was found to be well tolerated by bone marrow stromal cells^12^ was also well tolerated by primary PBMCs in the current study and its application permitted a reduction of the concentrations of carf. Indeed, our titration experiments in the two HMCLs L-363 and MM.1 S showed that already very low to moderate doses of carf which entailed no or at most moderate killing effects in MM cells when applied alone, strongly increased anti-myeloma toxicity when given in combination with VS-4718. In consequence, this drug combination might be specifically suitable for patients who are too fragile for high doses of carf, CAR T-cell therapy and other drugs with high toxicity and severe side effects^3,22^.

Moreover, the addition of VS-4718 allowed to overcome carf resistance in two cell line models with *PSMB5* mutations and PI triple-resistance^8^.Of note, both resistant HMCLs were clearly re-sensitized to carf upon addition of 3 µM VS-4718. Since this drug had little impact on the survival of non-tumor cells and was well tolerated in a pediatric pre-clinical testing program^14^, VS-4718 might thus not only be an alternative for fragile MM patients but also a suitable alternative for PI-resistant patients.

Furthermore, unlike other molecular inhibitors against e.g. BRAF that are limited to cases carrying e.g. a BRAFV600E mutation^23,24^, this study showed that VS-4718 affected viability and survival in all cell line models, irrespective of their molecular phenotype, suggesting that the effect on survival is not related to the described target molecules or any specific mutation profile.

Even though pPYK2 was not reduced and pFAK not measurable in L-363 and L-363R350Ixa cells after VS-4718 treatment, the impact on viability and survival was higher than for MM.1 S cells which showed a reduction in pPYK2 and pFAK levels upon treatment.

Thus, future studies might reveal additional VS-4718 targets and lead to a better understanding of the mechanistic background of the drug’s activity.

In conclusion, this study suggests that VS-4718 treatment has the potential to be an effective treatment with little side effects for most MM patients. It might also be of special value for fragile patients to reduce carf-associated toxicities and in clinical studies with relapsed/refractory PI-resistant patients to re-sensitize MM cells to carf. Further analyses, e.g. using primary MM cells and/or an animal MM model, are therefore warranted.

## Supplementary Information

Below is the link to the electronic supplementary material.

Supplementary Material 1

## Data Availability

Raw data from FACS analysis is provided upon request to the corresponding authors of this manuscript (ellen.leich@uni-wuerzburg.de; bruennert_d@ukw.de).
