# Supplementary material for: VS-4718 enhances apoptosis induced by low-dose carfilzomib and overcomes carfilzomib resistance in PSMB5-mutated proteasome inhibitor resistant multiple myeloma
Source: Sci Rep. 2026 Mar 16;16:9197. doi: 10.1038/s41598-026-43205-4 (PMC12996346; doi:10.1038/s41598-026-43205-4)

Supplementary Information

VS-4718 enhances apoptosis induced by low-dose carfilzomib and overcomes carfilzomib resistance in *PSMB5*-mutated proteasome inhibitor resistant multiple myeloma

Ellen Leich[1,#], Sofia Catalina Heredia-Guerrero[1,*], Marietheres Evers[1,*], Thorsten Stühmer[2,*], Tina Grieb[1], Hilka Rauert-Wunderlich[1], Ralf C. Bargou[2], Andreas Rosenwald[1], Manik Chatterjee[2], Daniela Brünnert[3,#]

[1] Institute of Pathology, University of Würzburg, Würzburg, Germany

[2] Comprehensive Cancer Center Mainfranken, University Hospital of Würzburg, Würzburg, Germany

[3] Experimental Tumor Immunology, Department of Obstetrics and Gynecology, University Hospital of Würzburg, Würzburg, Germany

*These authors contributed equally to this manuscript

#Corresponding authors

PD Dr. Ellen Leich-Zbat

University of Würzburg

Institute of Pathology

97080 Würzburg

Email: ellen.leich@uni-wuerzburg.de

Dr. Daniela Brünnert

University Hospital Würzburg

Experimental Tumor Immunology

Department of Obstetrics and Gynecology

Email: Bruennert_d@ukw.de

Figure S1

Expression and activation of PYK2 and FAK in seven human MM cell lines. Representative Western blots for three independent rounds. See uncropped blots below.

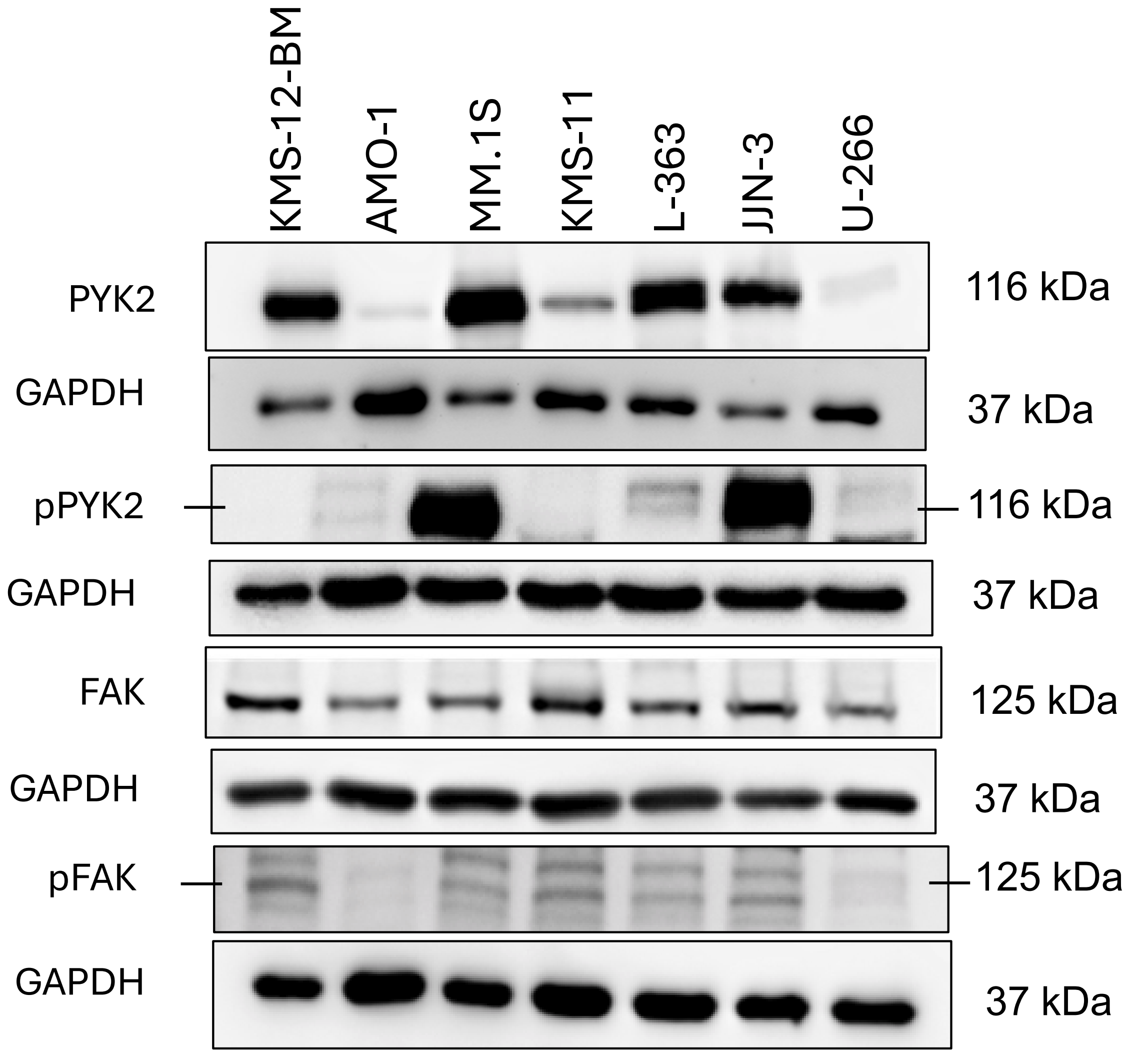

Figure S2

Titration of different VS-4718 concentrations in L-363. Bars represent the mean of three independent rounds ±SD. RM one-way ANOVA, Tukey's multiple comparison test.

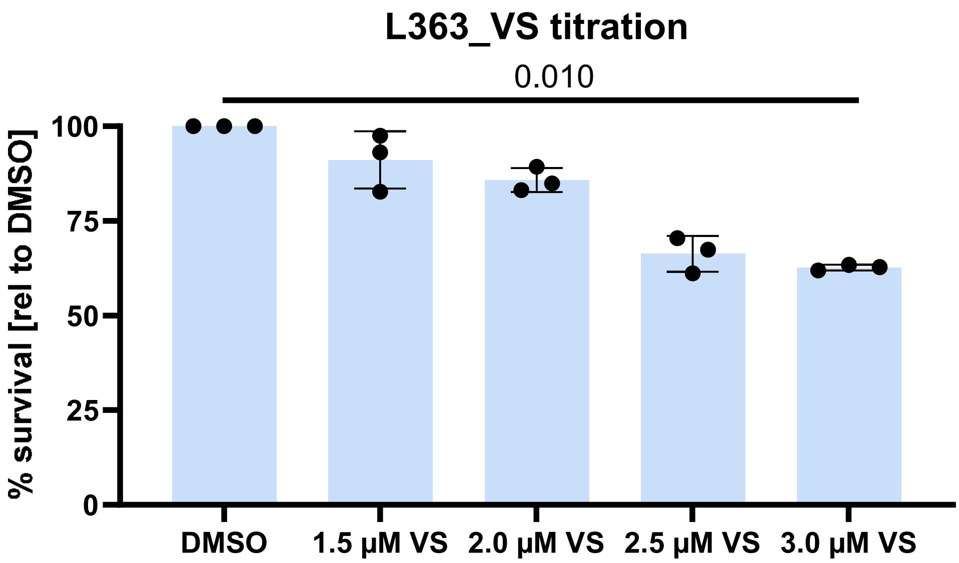

Figure S3
Test of different carf concentrations in parental and resistant L-363 and MM.1S cells. Concentrations used for Western blot experiments (Figure 5) are highlighted in red. Bars represent the mean of three indpendent rounds ±SD. *P*-values were determined using a multiple paired t-test.

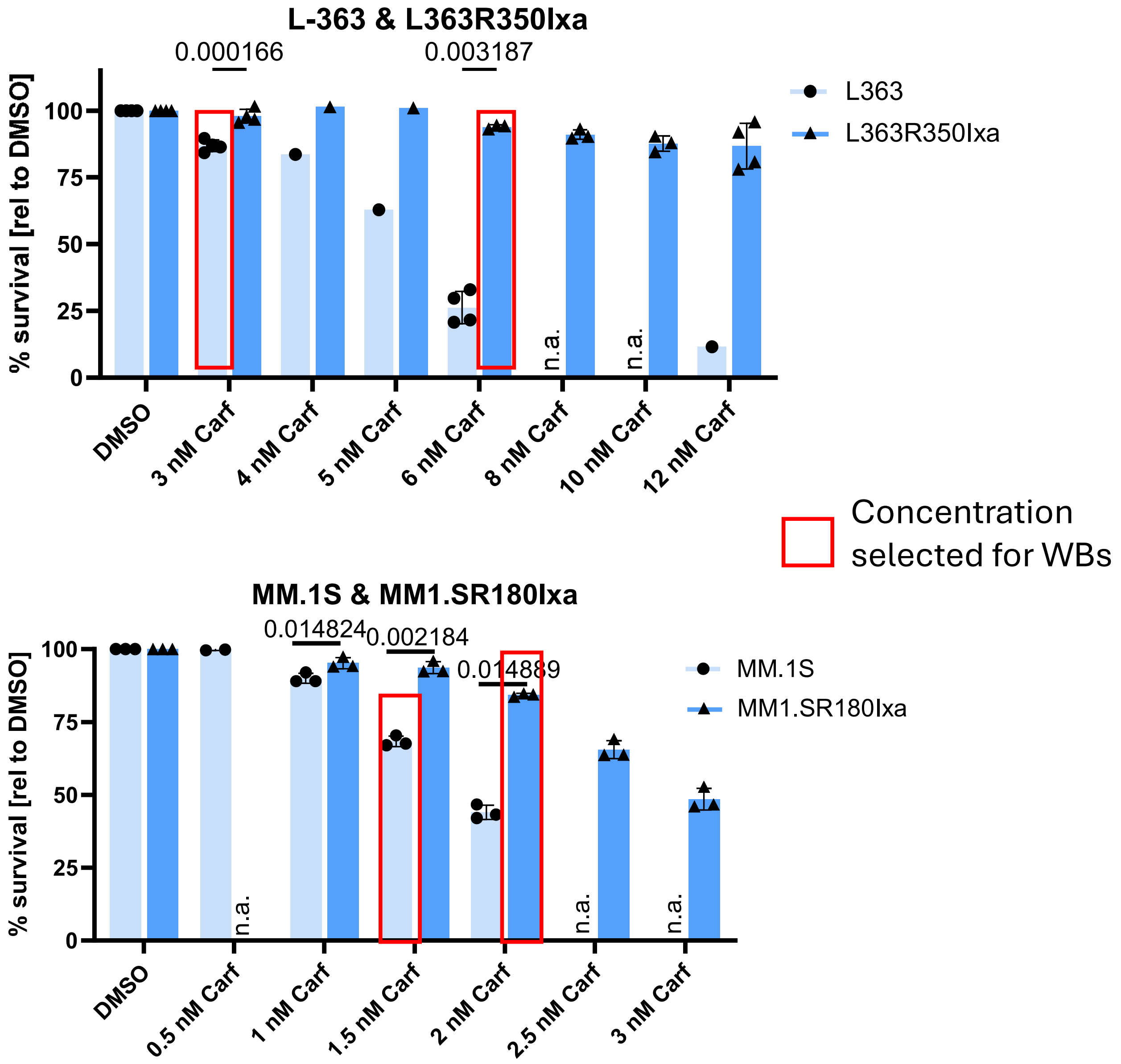

Figure S4

Western blot analysis to determine PARP-1 cleavage before and after inhibition with VS-4718 and carf alone and in combination. (**A**) L-363 cells were treated with 3 µM VS-4718 and 3 nM carf and L363R350Ixa cells with 3 µM VS-4718 and 6 nM carf for 24 h. (**B**) MM.1S cells treated with 3 µM VS-4718 and 1.5 nM carf alone and MM1.SR180Ixa cells with 3 µM VS-4718 and 2 nM carf for 24 h (**A, B**) Bars represent the mean ± SD of two independent experiments. Western blots from Figures 5 A and 5 B were stripped and then incubated with an antibody against PARP-1. For uncropped Western blots see section "uncropped Western blots" below.

A

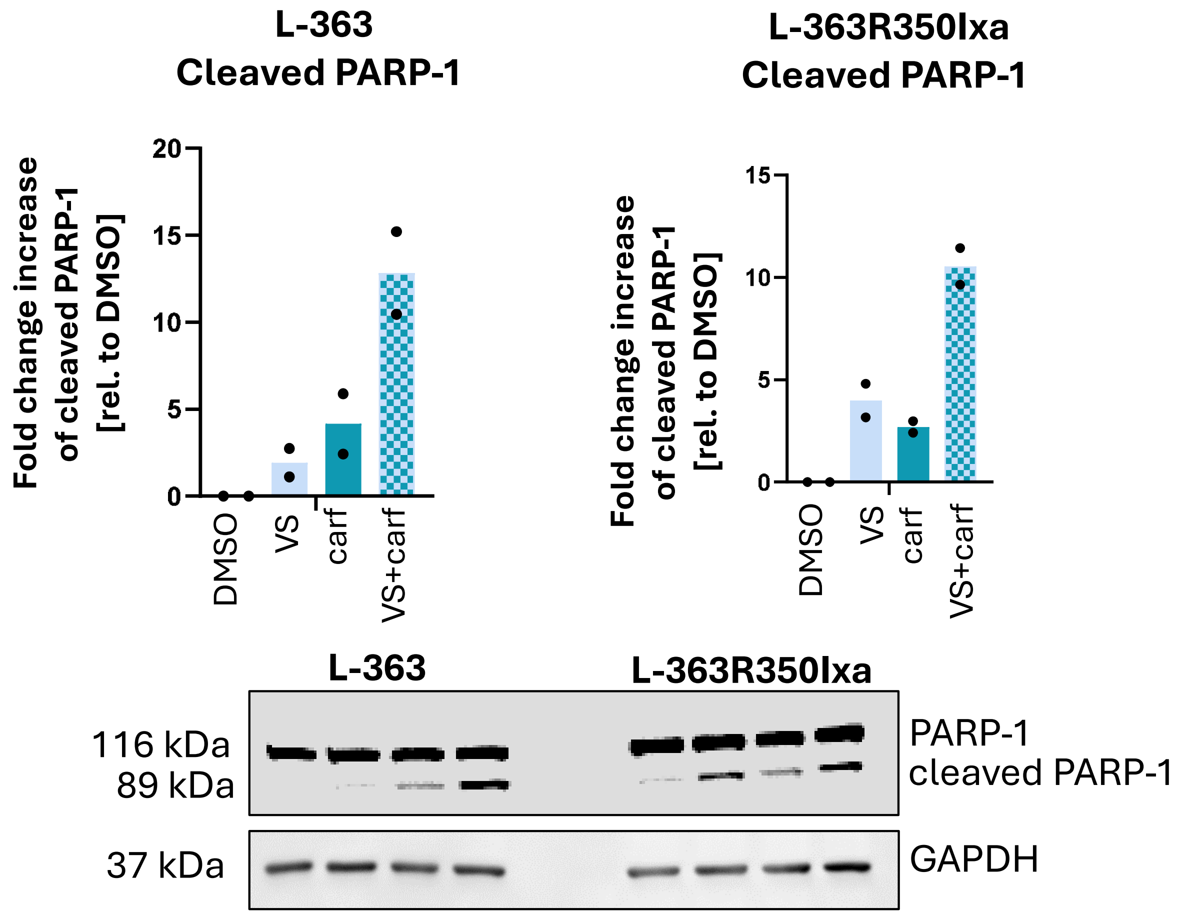

B

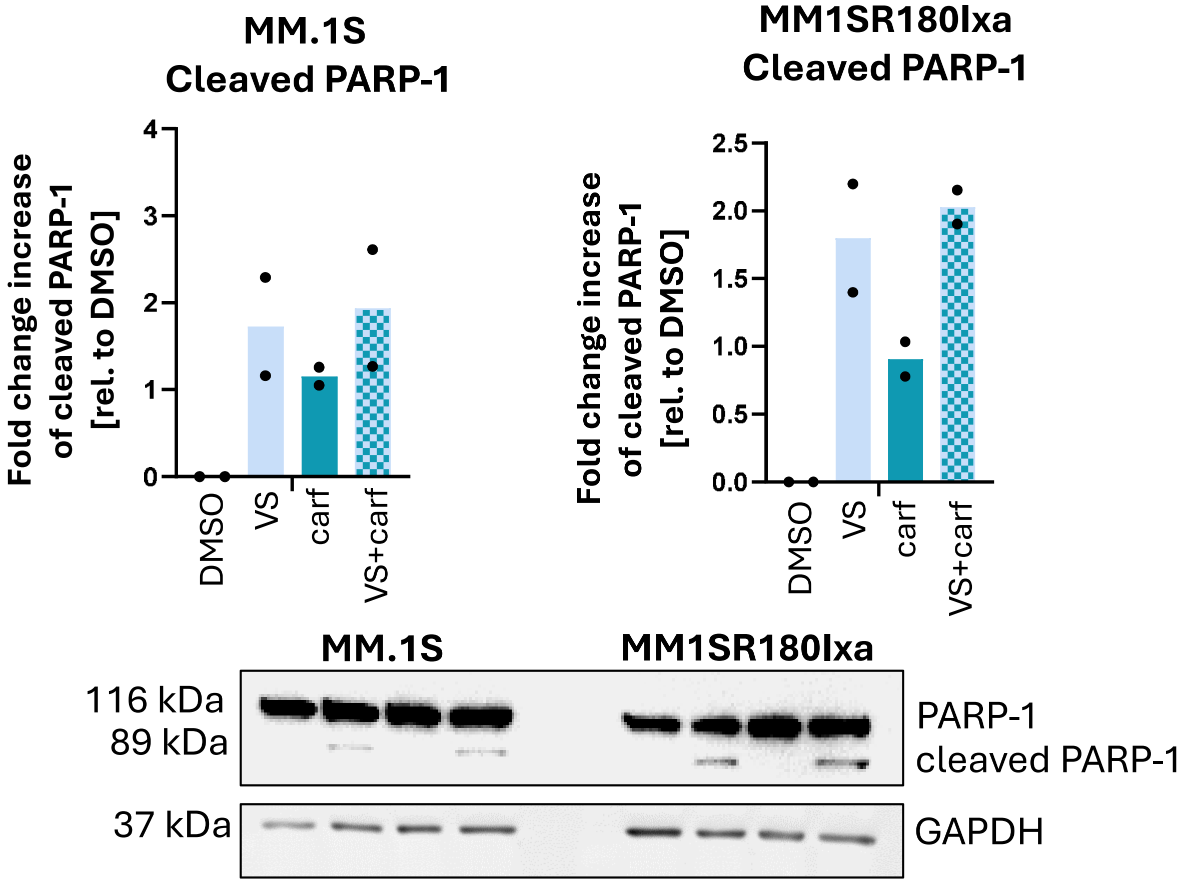

Uncropped Western blots

Uncropped Western blots with and without ladder of the experiment depicted in Figure 1 B

Treamtent of 7 HMCLs with VS-4718 (24h)

Order of loaded samples

KMS-12-BM
AMO-1
MM.1S
KMS-11
L-363
JJN-3
U-266

Detection of PARP-1

Houskeepers: β-Tubulin (55 kDa)
β-Actin (45 kDa)
GAPDH (37 kDa)

PageRuler™ Plus Prestained Protein Ladder, 10 to 250 kDa Katalognummer: #26619 Thermo Fischer

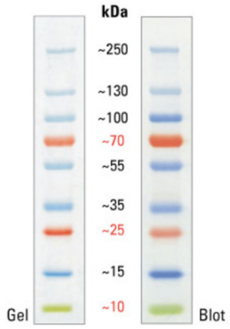

Round 1

PARP-1 (116 kDa)
Cleaved PAPR-1 (89 kDa)

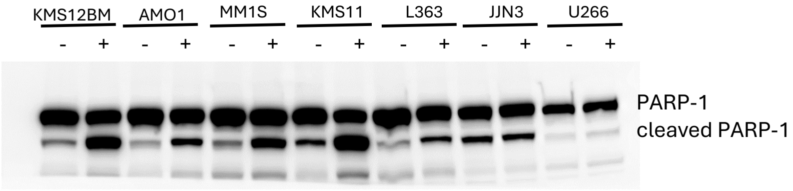

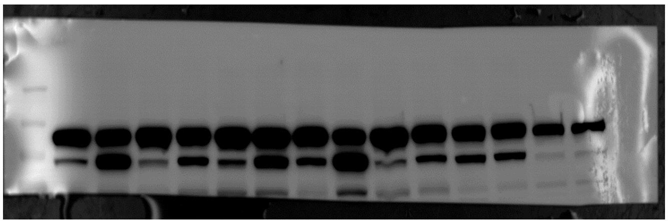

Housekeepers

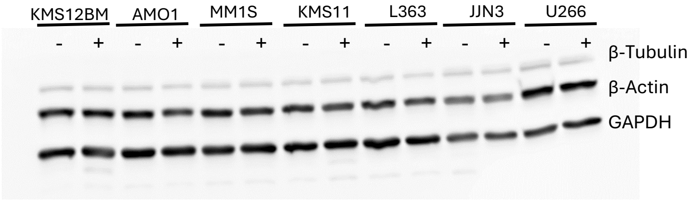

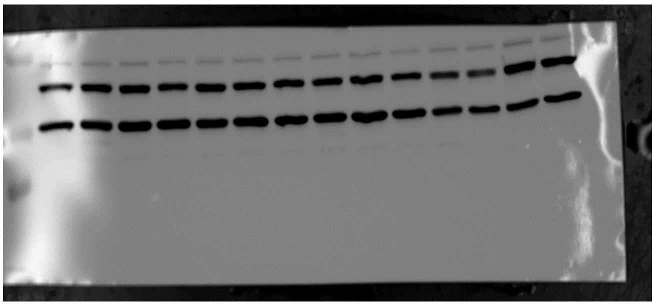

Round 2

PARP-1 (116 kDa)
Cleaved PAPR-1 (89 kDa)

Housekeepers

β-Tubulin (55 kDa)
β-Actin (45 kDa)
GAPDH (37 kDa)

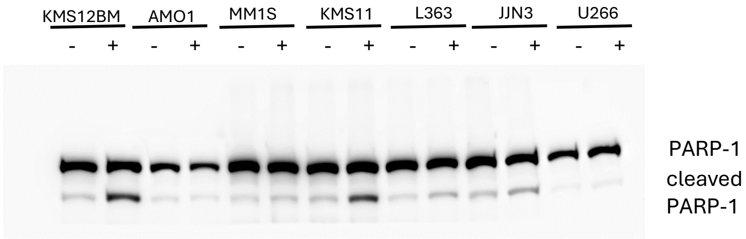

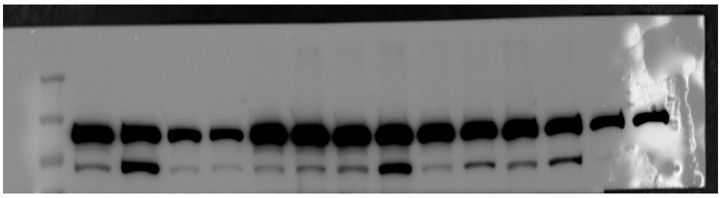

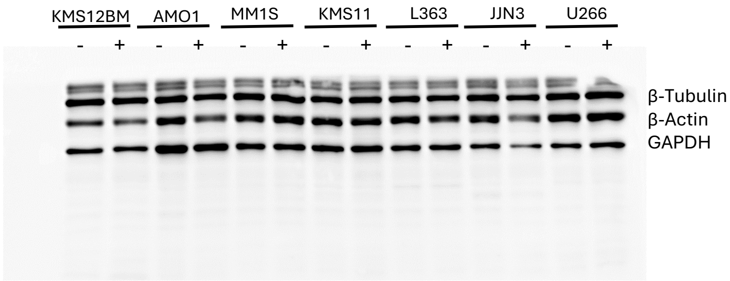

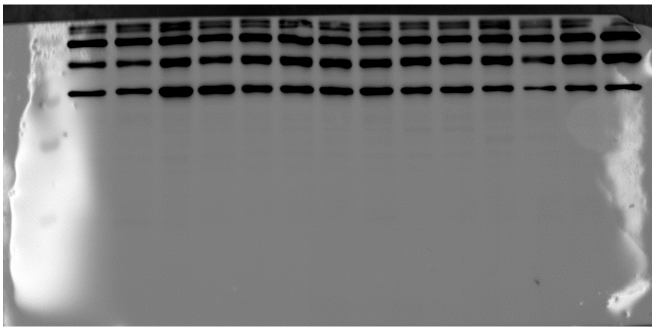

Round 3

PARP-1 (116 kDa)
Cleaved PAPR-1 (89 kDa)

Housekeepers

β-Tubulin (55 kDa)
β-Actin (45 kDa)
GAPDH (37 kDa)

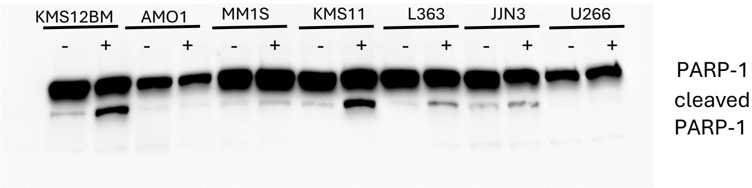

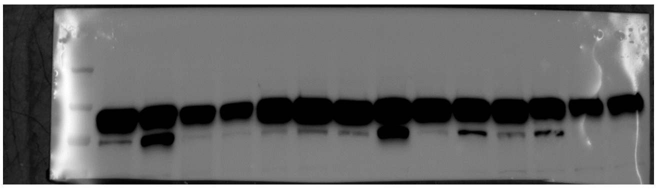

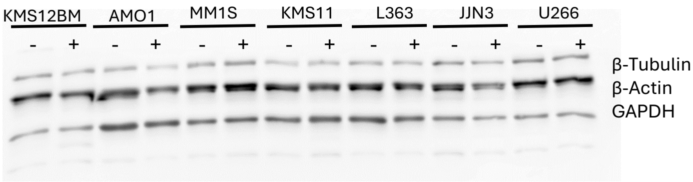

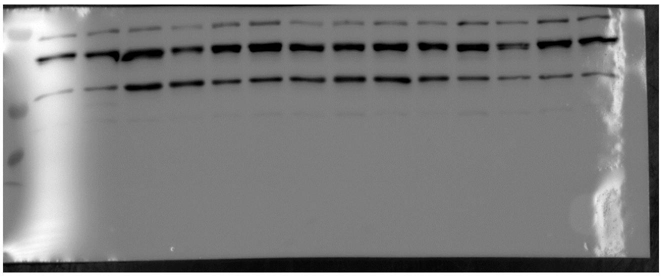

Uncropped Western blots depicted in Figure 5 A

Order of loaded samples

MM1S: DMSO VS3 CARF1.5 V+C (lane 2-5)

MM1S IXA: DMSO VS3 CARF2 V+C (lane 8-11)

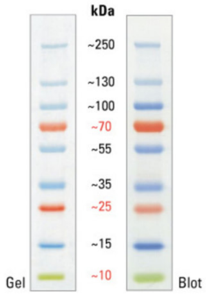

FAK + corresponding GAPDH
(**Same GAPDH blot as shown along with the corresponding uncropped PARP-1 blot to Figure S4 B.**)

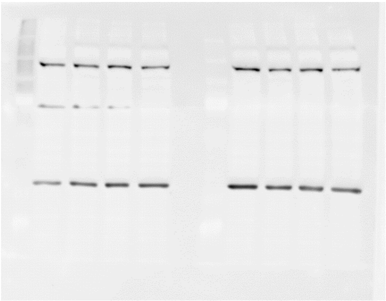

FAK (125 kDa)

GAPDH (37 kDa)

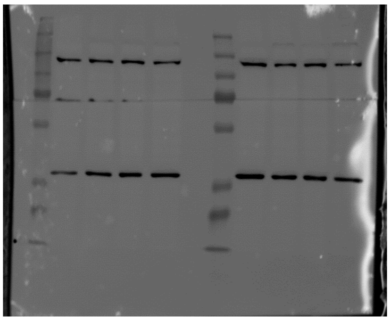

FAK (125 kDa)

GAPDH (37 kDa)

pFAK + corresponding GAPDH

pFAK (125 kDa)

GAPDH 37 kDa)

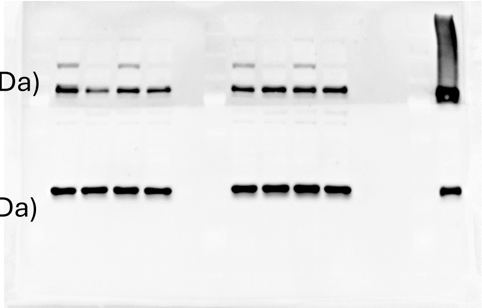

pFAK (125 kDa)

GAPDH 37 kDa)

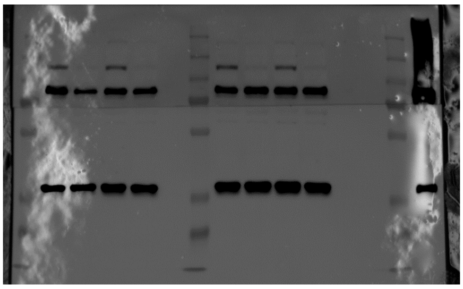

PYK2 + corresponding GAPDH

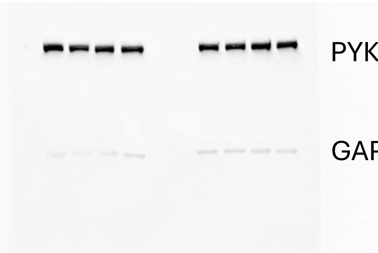

PYK2 (116 kDa)

GAPDH (37 kDa)

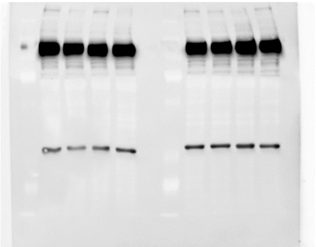

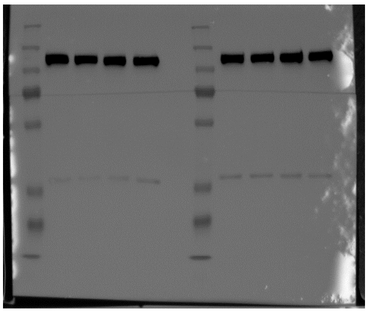

PYK2 (116 kDa)

GAPDH (37 kDa)

pPYK2 + corresponding GAPDH

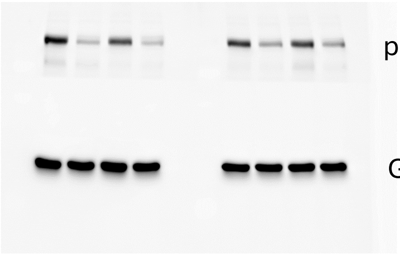

pPyk2 116 kDa)

GAPDH (37 kDa)

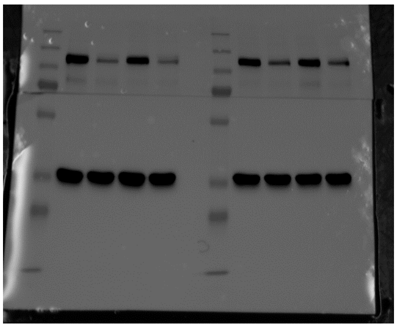

pPYK2 (116 kDa)

GAPDH (37 kDa)

Uncropped Western blots depicted in Figure 5 B

Order of loaded samples

L363: DMSO VS3 CARF3 V+C (lane 2-5)
L363 IXA: DMSO VS3 CARF6 V+C (lane 8-11)

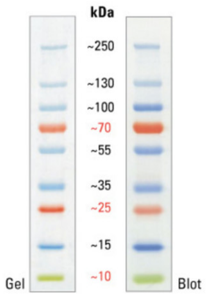

FAK + corresponding GAPDH

(**Same GAPDH blot as shown along with the corresponding uncropped PARP-1 blot to Figure S4 A.**)

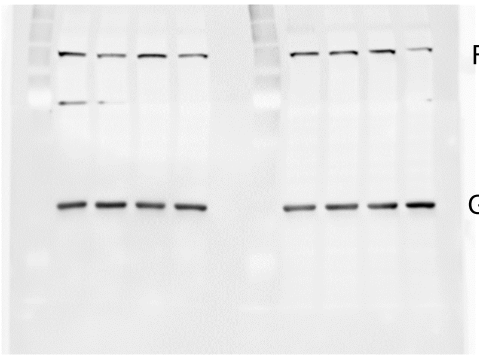

FAK (125 kDa)

GAPDH (37 kDa)

pFAK + corresponding GAPDH

pFAK (125 kDa)

GAPDH (37 kD

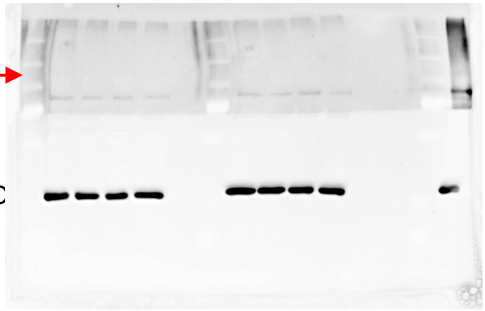

PYK2 + corresponding GAPDH

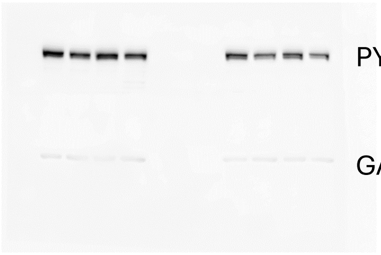

PYK2 (116 kDa)

GAPDH (37 kDa)

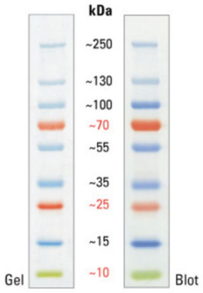

pPYK2 + corresponding GAPDH

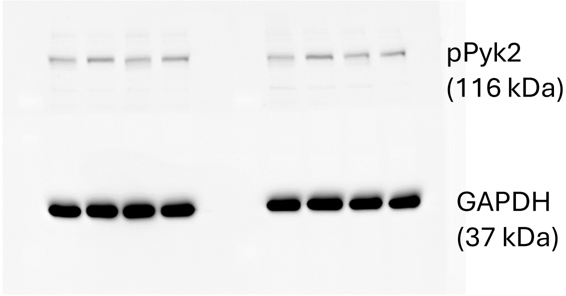

pPyk2 (116 kDa)

GAPDH (37 kDa)

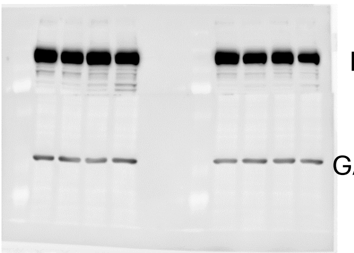

PYK2 (116 kDa)

GAPDH (37 kDa)

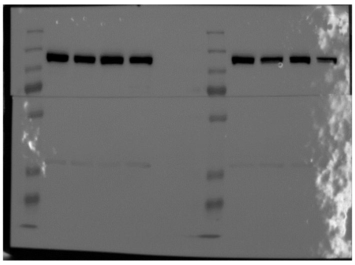

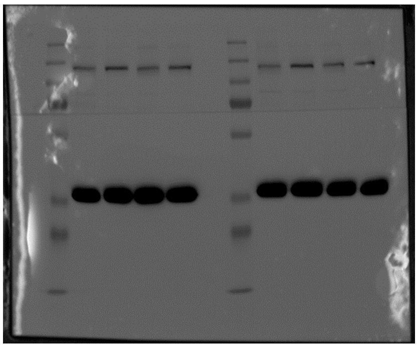

Uncropped Western blots depicted in Figure S1

Order of loaded samples

KMS-12-BM

AMO-1

MM.1S

KMS-11

L-363

JJN-3

U-266

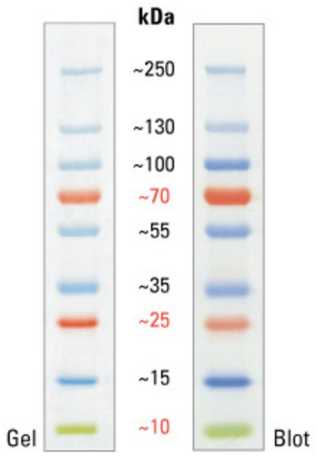

Detection of:
PYK2
pPYK2
FAK
pFAK

Housekeeper:
GAPDH

PYK2

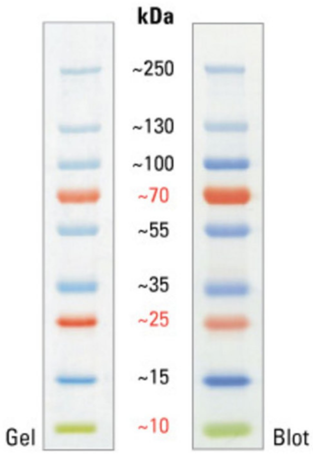

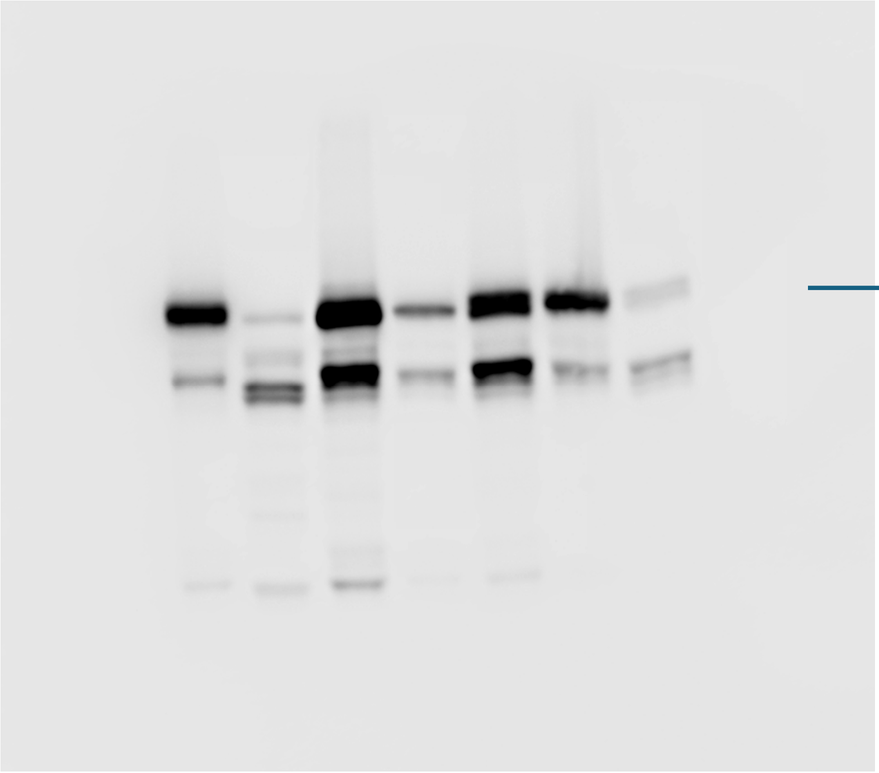

PYK2 (116 kDa)

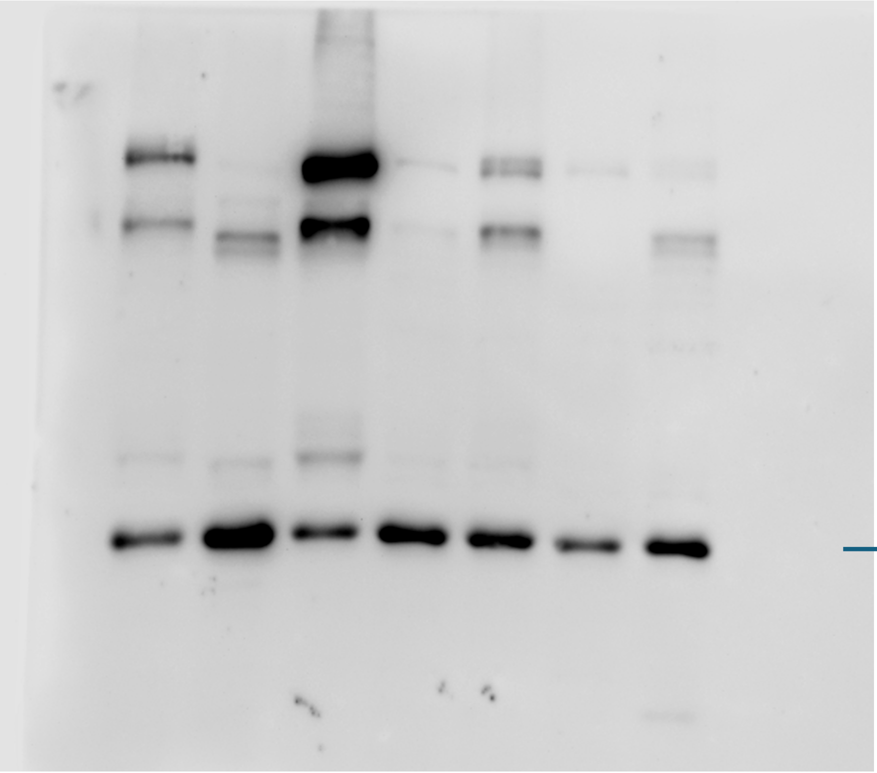

GAPDH (37 kDa)

pPYK2

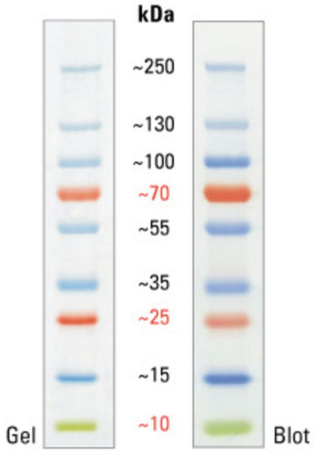

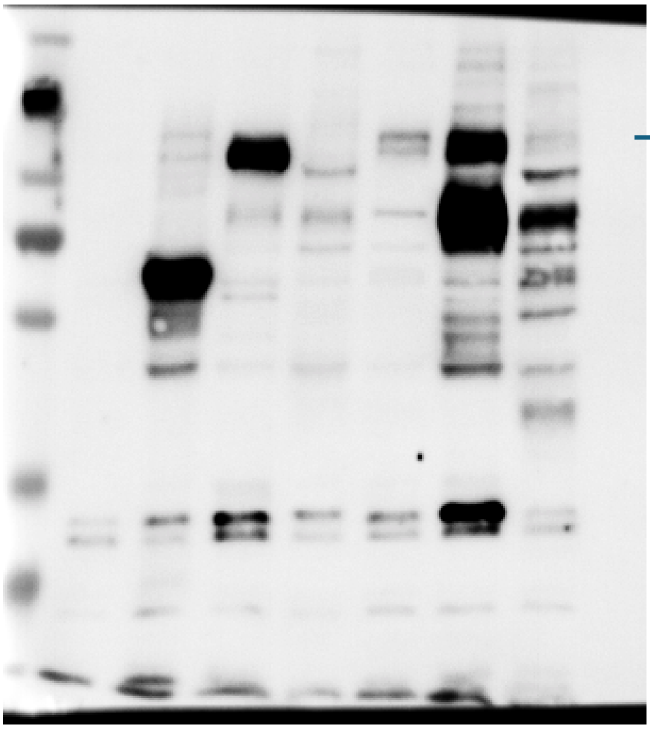

pPYK2 (116 kDa)

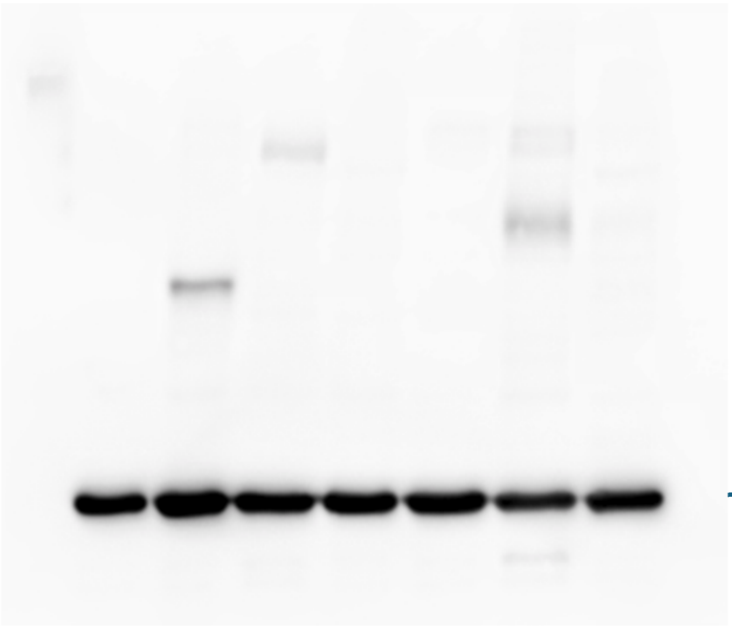

GAPDH (37 kDa)

FAK

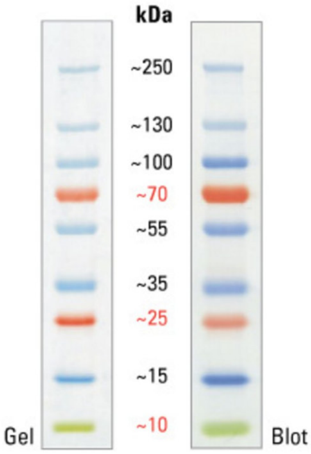
kDa
~250
~130
~100
~70
~55
~35
~25
~15
~10
Gel
Blot

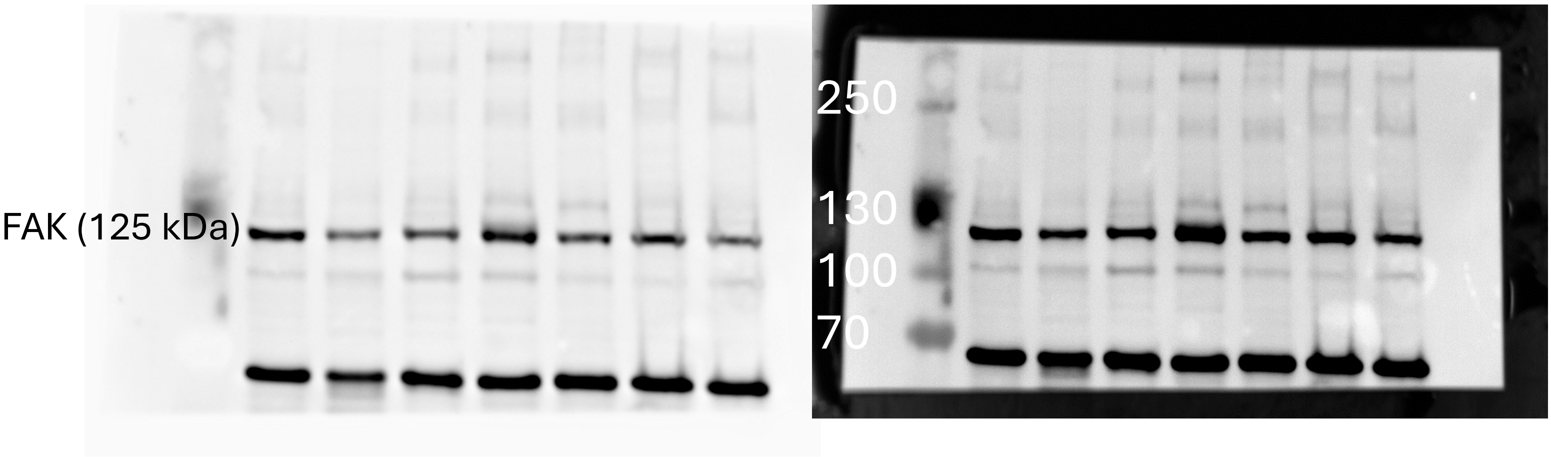
FAK (125 kDa)
250
130
100
70

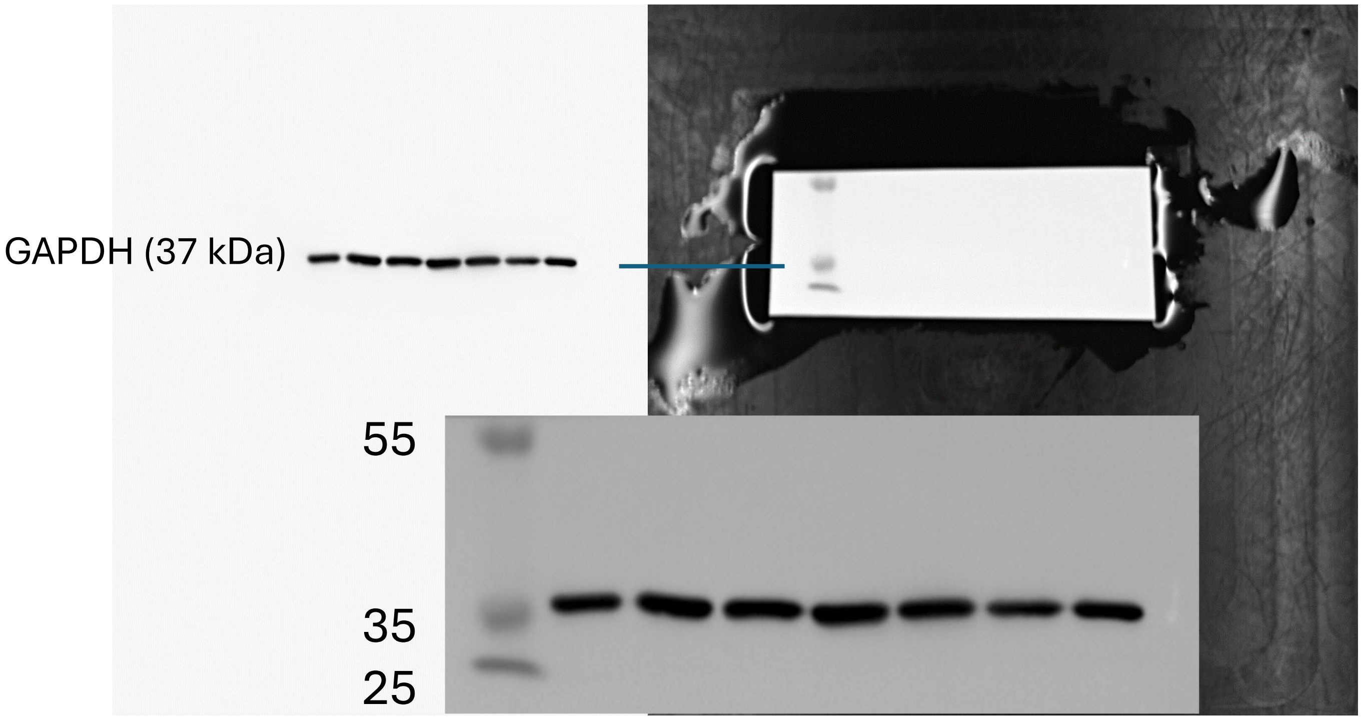
GAPDH (37 kDa)
55
35
25

pFAK

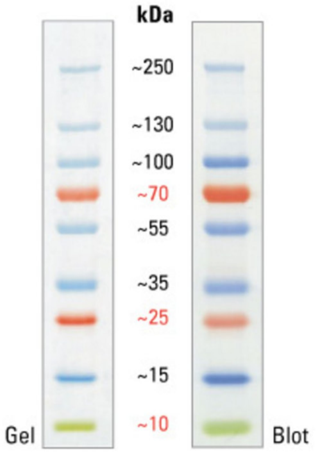

pFAK
(125 kDa)

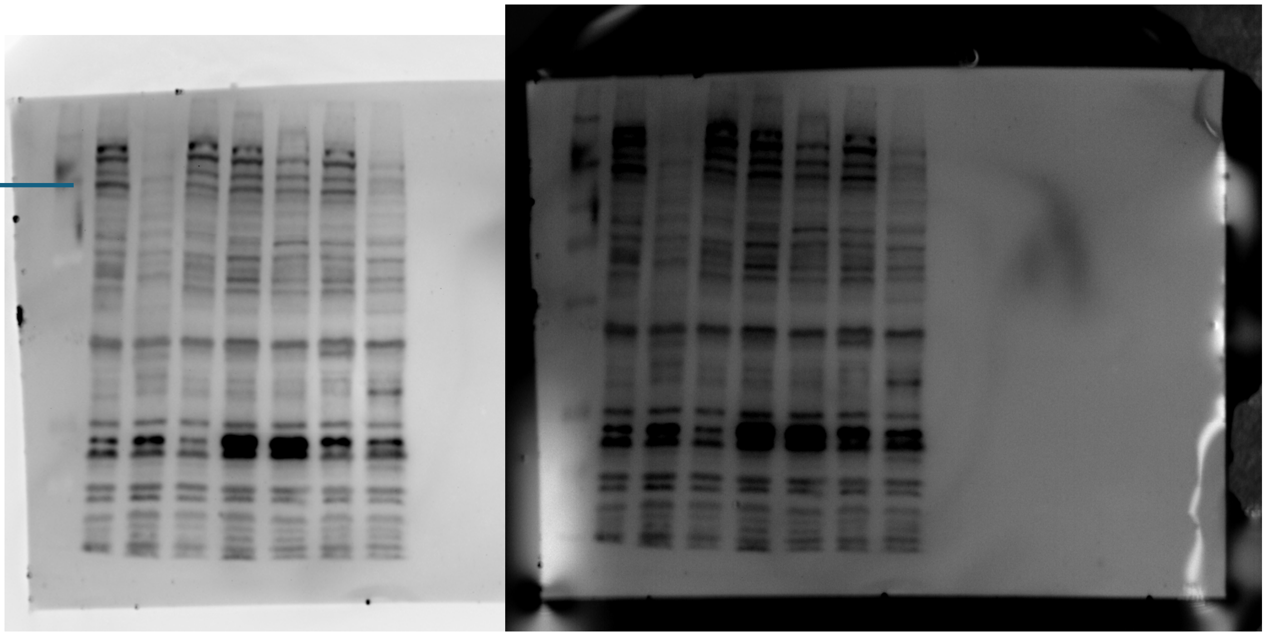

GAPDH (37 kDa)

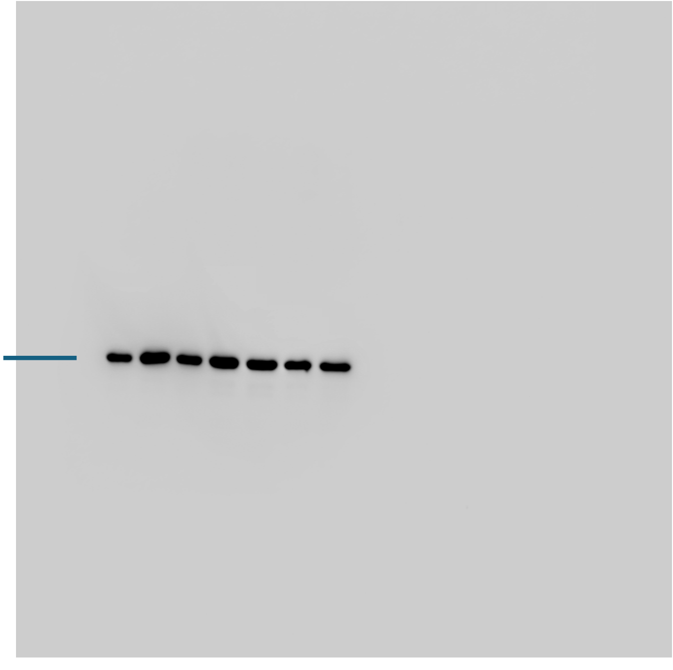

Uncropped stripped Western blots hybridized with PARP-1 depicted in Figure S4

PARP-1 (116 kDa), Cleaved PARP-1 (89 kDa)

A

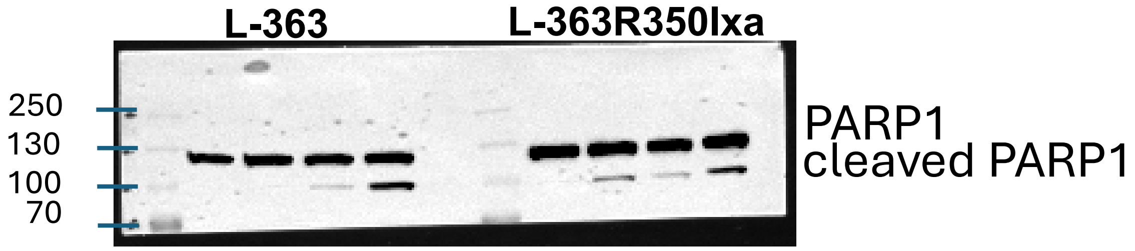

FAK + corresponding GAPDH
(**Same GAPDH blot as shown along with the corresponding uncropped FAK blot to Figure 5B.**)

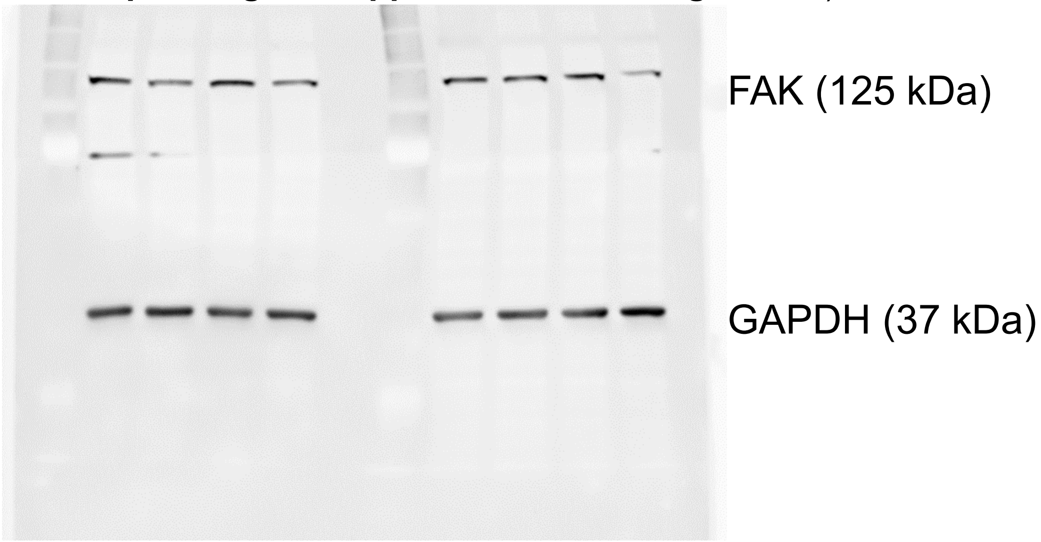

B

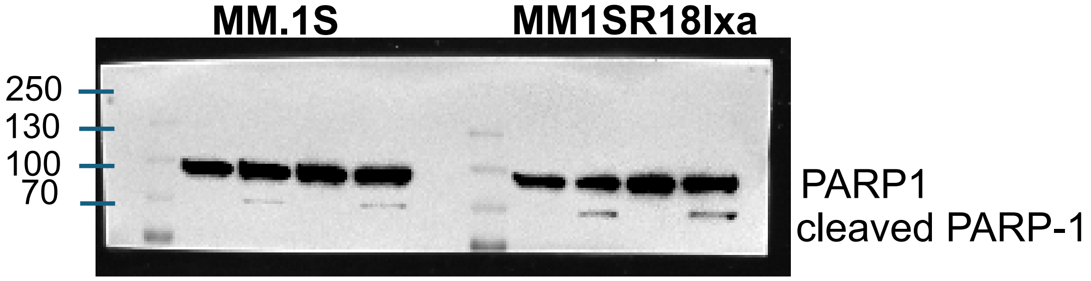

FAK + corresponding GAPDH
(**Same GAPDH blot as shown along with the corresponding uncropped FAK blot to Figure 5A.**)

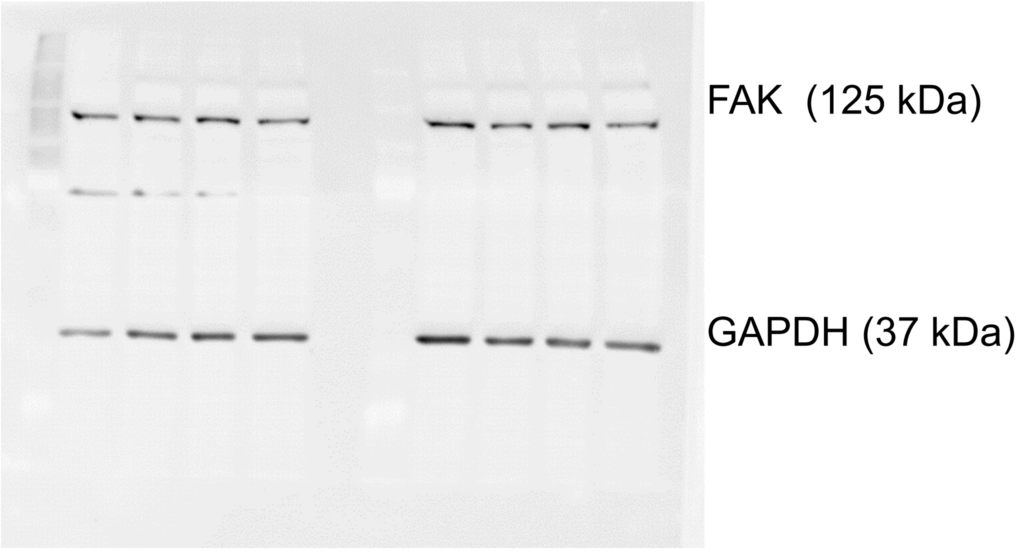

Supplement: Supplementary file 1 — Supplementary Material 1 [file 41598_2026_43205_MOESM1_ESM.pdf]
